# Unsteady MHD natural convection flow of Casson fluid incorporating thermal radiative flux and heat injection/suction mechanism under variable wall conditions

**DOI:** 10.1038/s41598-021-83691-2

**Published:** 2021-02-19

**Authors:** Talha Anwar, Poom Kumam, Wiboonsak Watthayu

**Affiliations:** 1 Department of Mathematics, Faculty of Science, King Mongkut’s University of Technology Thonburi (KMUTT), 126 Pracha-Uthit Road, Bang Mod, Thung Khru, Bangkok, 10140 Thailand; 2 KMUTT Fixed Point Research Laboratory, Room SCL 802 Fixed Point Laboratory, Science Laboratory Building, Department of Mathematics, Faculty of Science, King Mongkut’s University of Technology Thonburi (KMUTT), 126 Pracha-Uthit Road, Bang Mod, Thung Khru, Bangkok, 10140 Thailand; 3 Center of Excellence in Theoretical and Computational Science (TaCS-CoE), Science Laboratory Building, Faculty of Science, King Mongkut’s University of Technology Thonburi (KMUTT), 126 Pracha-Uthit Road, Bang Mod, Thung Khru, Bangkok, 10140 Thailand; 4 Department of Medical Research, China Medical University Hospital, China Medical University, Taichung, 40402 Taiwan

**Keywords:** Applied mathematics, Electrical and electronic engineering

## Abstract

Unsteady magnetohydrodynamic flow of Casson fluid over an infinite vertical plate is examined under ramped temperature and velocity conditions at the wall. Thermal radiation flux and heat injection/suction terms are also incorporated in the energy equation. The electrically conducting fluid is flowing through a porous material and these phenomena are governed by partial differential equations. After employing some adequate dimensionless variables, the solutions are evaluated by dint of Laplace transform. In addition, the physical contribution of substantial parameters such as Grashof number, radiation parameter, heat injection/suction parameter, porosity parameter, Prandtl number, and magnetic parameter is appropriately elucidated with the aid of graphical and tabular illustrations. The expressions for skin friction and Nusselt number are also derived to observe wall shear stress and rate of heat transfer. A graphical comparison between solutions corresponding to ramped and constant conditions at the wall is also provided. It is observed that graphs of the solutions computed under constant conditions are always superior with respect to graphs of ramped conditions. The magnetic field decelerates the flow, whereas the radiative flux leads to an upsurge in the flow. Furthermore, the shear stress is a decreasing function of the magnetic parameter.

## Introduction

The fluid is a specific type of matter which easily goes under deformation when an external force is applied, and it has no particular shape^1^. Mainly, fluids are partitioned as Newtonian fluid and non-Newtonian fluids. Non-Newtonian fluids have numerous practical and industrial applications, and such fluids involve honey, blood, greases, oils, and foodstuff^2^. Polymer industries, textile, irrigation problems, and biological systems incorporate flows of non-Newtonian fluids in porous medium encountering magnetic effects. Moreover, free and forced convection flows of non-Newtonian fluids together with magnetohydrodynamic (MHD) have a wide range of applications in polymer fabrication, MHD pumps and motors, aerodynamic heating, and purification of mineral oil. Thermal engineering and welding mechanics involve the addition of heat injectors or sinks to the aforementioned flows for heating and cooling processes^3,4^. To forecast the response of non-Newtonian fluids in different kinds of reservoirs, hydrologists studied and discussed their flows in porous media ranging from sand packs to fused Pyrex glasses^5^. In metallurgy, for the solidification process, a magnetic field is imposed on a liquid metal, which flows through a porous material^6^. Convective flows of non-Newtonian fluids have a key role in the agriculture field to discover sub-ground water reservoirs^7^. In addition, flows of non-Newtonian fluids incorporating radiative flux are of prime use in the mechanisms of polymeric mixtures, aerosol technology, and solar collectors which operate at very low and high temperatures^8^.

In order to anticipate and optimize the effectiveness of the aforementioned practical phenomena, a wide range of studies is conducted and discussed by many researchers. Petrovic et al.^9^ studied MHD flow and heat transfer for two immiscible fluids in a porous medium. Ibrahim et al.^10^ discussed the effects of a heat source and Joule heating on MHD radiative flow over a porous stretching sheet. Entropy generation for compressible radiative MHD fluid flow in a channel partially filled with porous material was reported by Jain et al.^11^. Hasnain et al.^12^ provided solutions for MHD two-phase mixed convection fluid flow in an inclined channel filled with porous medium. Pattnaik et al.^13^ derived analytic solutions for natural convection flow with porosity and MHD effects by inculcating time-dependent concentration and temperature at the boundary. Velocity and heat transfer phenomena for micropolar MHD flow over a vertically moving plate immersed in porous media were interpreted by Kim^14^. The influence of heat injection and suction on flow under the effect of an imposed magnetic field between two concentric porous cylinders was reported by Hamza^15^.

Casson fluid model was initially proposed by Casson in 1959 for the anticipation of flow trends of suspended pigment-oil^16^. In the case of small shear stress, Casson fluid behaves like an elastic solid and no flow takes place. On the other hand, the dominance of the shear stress magnitude of Casson fluid against yield shear stress ensures the flow of Casson fluid. This fluid is based on a structural model of bilateral behavior of liquid and solid phases of two-phase suspension. Some significant examples of Casson fluid are honey, jelly, soup, concentrated fruit juices, and artificial fibers. The participation of Casson fluid can be observed in the preparation of multiple products such as synthetic lubricants, pharmaceutical chemicals, paints, coal, tomato sauce, china clay, and many others. Since human blood is comprised of various substances like fibrinogen, human red blood cells, protein, and globulin in aqueous base plasma, it can also be considered as Casson fluid^17,18^. A wide variety of researchers have been fascinated by efficacious applications of Casson fluid in drilling processes, biological treatments, food processing, and bio-engineering operations. Khalid et al.^19^ investigated the time-dependent free convectional MHD flow of Casson fluid in a porous material. The flow of Casson fluid through tubes was initially discussed by Oka^20^. MHD Casson fluid flow over a shrinking/stretching surface was examined by Bhattacharyya et al.^21^. Mernone et al.^22^ explained the two-dimensional peristaltic flow of Casson fluid in a channel. Mustafa et al.^23^ implicated the homotopy analysis method to provide an analysis of heat transfer and time-dependent boundary layer flow of the Casson model over a flat moving plate. Impacts of heat blowing/suction and thermal radiation on temperature and flow of Casson fluid over stretching sheet were analyzed by Mukhopadhyay^24^. Pramanik^25^ conducted a systematic study to evaluate the impacts of porosity and radiative heat flux on heat and mass transfer. Arthur et al.^26^ discussed the influence of chemical reaction and magnetic field on the flow of Casson fluid past a porous perpendicular surface.

The transportation of heat transferring fluids exhibits a significant role in widespread industrial and engineering operations, for example, thermal management of high-tech systems, designing of devices, cooling and heating processes, manufacturing of gas turbines, nuclear operations, and so forth. However, investigations for unsteady MHD flows of non-Newtonian fluids in porous mediums subjected to ramped velocity and ramped temperature conditions simultaneously are very few in the literature because it is intricate to handle the resulted nonlinear complex relations analytically though, these conditions have significant practical utilities. For instance, ramped velocity is an efficient aid to develop prognoses, determining treatment, and examining heart and blood vessels. Additionally, diagnoses and establishing treatment of cardiovascular deceases through treadmill testing and ergometry also depend on ramped velocity^27^. The idea of ramped velocity and ramped temperature condition was initiated by Ahmed and Dutta to examine the unsteady flow of Newtonian fluid moving over a vertically infinite wall in the existence of mass transfer^28^. Bruce^29^, and Myers and Bellin^30^ further evaluated the contribution of ramped velocity in treadmill testing. Malhotra et al.^31^, Schetz^32^, and Hayday^33^ have introduced the ramped wall temperature condition. One of the most vital appeals of ramped wall temperature is to demolish the cancerous cells through thermal therapy. Kundu^34^ provided five kinds of thermal boundary conditions to obtain the desired results of cancer treatment, adjacently, minimizing the side effects of thermal therapy to almost nonexistence. A comprehensive comparison and survey report^35^ describe that ramped heating is an efficient support to control the rise in temperature occurring because of adiabatic condition, in the chemical industry. Das et al.^36^ evaluated the influence of ramped temperature condition on incompressible optically thin fluid flow over an impulsively upright plate. Nandkeolyar et al.^37^ studied and compared various kinds of plate’s movement having periodic acceleration, single acceleration, and uniform velocity subjected to constant wall and ramped wall conditions for natural convective flows of viscous fluids in the existence of magnetic field. A detailed analysis of several physical phenomena of Hall current, thermal radiation, Darcy’s law, heat consumption, and chemical reaction on mass and heat transfer with ramped wall concentration and temperature subjected to impulsive and accelerating upright plates was provided by Seth et al.^38–40^. Chandran et al.^41^ presented the impact of ramped wall temperature on convective viscous fluid flow, which was later extended by Seth et al.^42^ for the porous medium. Zin et al.^43^ investigated the effects of thermal radiation and magnetic field on free convection Jeffrey fluid flow with ramped wall temperature. This study was further extended for simultaneous application of ramped boundary conditions (ramped wall velocity and ramped wall temperature) by Maqbool et al.^44^. Tiwana et al.^45^ recently studied the MHD time-dependent convective flow of Oldroyd-B fluid considering simultaneous ramped conditions at the boundary.

However no investigation for Casson fluid flow with ramped wall velocity and ramped wall temperature conditions is yet available in the literature. To effectively fill this gap, this study comprises of unsteady incompressible MHD flow of Casson fluid, subjected to ramped velocity and ramped temperature conditions at a vertical wall, which is placed in the porous medium. Additionally, radiative flux and heat injection/suction are also inculcated in the heat transfer mechanism to evaluate their significance. Laplace transformation and the Durbin method are implemented to execute the solutions of modeled momentum and energy equations. Finally, the physical features of relevant parameters are analyzed with the help of tables and graphs.

## Mathematical modeling of the problem

**Figure 1:**
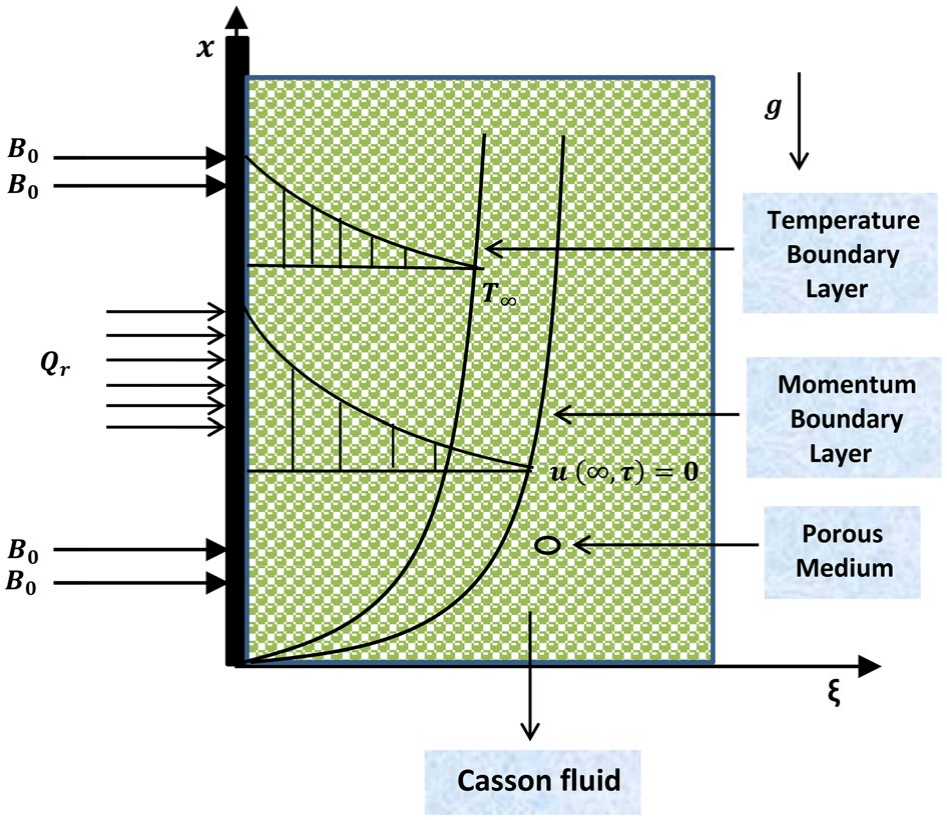
Geometry of the considered model.

We assume Casson fluid flow over an infinite vertical plate saturated in a porous medium. The plate is considered at $\xi =0$ and flow is restricted to $\xi >0$, which is along the direction of the plate (as depicted in Fig. 1). To govern the model, the following key assumptions are taken into account

- The flow is one-dimensional and unidirectional.
- A uniform magnetic field of magnitude $B_0$ is imposed perpendicular to the direction of the plate.
- Reynolds number is small enough to ignore the effect of an induced magnetic field.
- The radiative heat flux $Q_r$ along the direction of the plate is negligible against the radiative heat flux normal to the plate.
- In order to ignore the polarization effect of fluid, no electric field is applied.
- The temperature equation is free from viscous dissipation term.

The family of Maxwell equations is presented to deal with the magnetic field

1 $${\text{Curl}} \mathbf{B} = {\mu _m} \mathbf{J},\;\;{\text{Curl}} \mathbf{E} = - \frac{{\partial {\mathbf{B}}}}{{\partial t}},\;\;{\text{div}}\mathbf{B} = 0,$$

where $\mu _m$ denotes magnetic permeability, ${\mathbf {J}}$ denotes current density, and ${\mathbf {E}}$ denotes electric field. Furthermore, ${\mathbf {B}}$ is the summation of imposed magnetic field ($B_0$) and induced magnetic field ($b_0$), which is ignored in this case. From Ohm’s law

2 $$\begin{aligned} {\mathbf {J}} = ({\mathbf {V}} \times {\mathbf {B}} +{\mathbf {E}}) \sigma , \end{aligned}$$

where $\sigma$ is the electrical conductivity and ${\mathbf {V}}$ is the velocity of fluid. Furthermore, the supposition of a small Reynolds number leads to

3 $$\begin{aligned} (\mathbf {J} \times \mathbf {B})\frac{1}{\rho }=[(\mathbf {V} \times \mathbf {B}_\mathbf {0}) \times \mathbf {B}_\mathbf{0}] \frac{\sigma }{\rho }=-\frac{\sigma B^2_0 u}{\rho }. \end{aligned}$$

For an incompressible Casson fluid, Cauchy stress tensor for rheological state takes the following form^16,46,47^

4 $$\begin{aligned} \uptau _{xy}=\left\{ \begin{array}{ll} 2 \left( \mu _{\lambda }+\frac{P_\eta }{\sqrt{2 \pi }} \right) e_{xy} \quad \pi > \pi _c \\ 2 \left( \mu _{\lambda }+\frac{P_\eta }{\sqrt{2 \pi _c}} \right) e_{xy} \quad \pi < \pi _c \end{array}\right. , \end{aligned}$$

where $P_\eta$ is yielded stress, $\mu _\lambda$ is the plastic dynamic viscosity, $\pi$ is the self product of the component of deformation rate, $\pi _c$ is the critical value of earlier mentioned product depending upon the non-Newtonian model, and $e_{xy}$ is the $(x,y)^{xh}$ component of the rate of deformation. Initially, at time $\tau =0$, both plate and fluid are static with uniform temperature $T_\infty$. For time $\tau >0$, ramped boundary conditions for both velocity and temperature are considered at the wall $(\xi =0)$ such that up to some characteristic time $\tau _0$, both velocity and temperature depend upon the fraction of time $\tau$ and characteristic time $\tau _0$, and later they take uniform values. Mathematically

5 $$\begin{aligned} u(0,\tau )&=\left\{ \begin{array}{ll} u_c\frac{\tau }{\tau _0} \quad 0 < \tau \le \tau _0 \\ u_c \qquad \qquad \tau >\tau _0 \end{array}\right. , \qquad \qquad \qquad \end{aligned}$$

6 $$\begin{aligned} T(0,\tau )&=\left\{ \begin{array}{ll} T_\infty +(T_w-T_\infty )\frac{\tau }{\tau _0} \quad 0 < \tau \le \tau _0 \\ T_w \qquad \qquad \qquad \qquad \qquad \tau >\tau _0 \end{array}\right. , \end{aligned}$$

where $u_c$ is characteristic velocity, *u* is a component of velocity along *x*-axis, $T_w$ is wall temperature, and $T_\infty$ is constant ambient temperature. Since we have considered ramped wall velocity and ramped wall temperature at the same time, this can be regarded as a simultaneous application of ramped boundary conditions. For Casson fluid, first time such boundary conditions are considered though they have wide applications in industrial and medical sciences. On the basis of all aforementioned assumptions, we obtain the following principal equations for flow and energy transfer^19,48^:

7 $$\begin{aligned} \rho \frac{\partial u}{\partial \tau }=&\mu \left( 1+\frac{1}{\alpha } \right) \frac{\partial ^2 u}{\partial \xi ^2}-\sigma B_0^2 u + \rho g \beta (T-T_\infty )-\left( 1+\frac{1}{\alpha } \right) \frac{\mu \phi }{k_p}u, \end{aligned}$$

8 $$\begin{aligned} \rho c_p \frac{\partial T}{\partial \tau }=&k\frac{\partial ^2 T}{\partial \xi ^2}-\frac{\partial Q_r}{\partial \xi }+Q_0 (T-T_\infty ), \end{aligned}$$

where $\alpha =\mu \sqrt{2 \pi _c}/P_\eta$ is the parameter of Casson fluid, $\rho$ is the fluid density, *g* is gravitational pull, $\beta$ is thermal expansion coefficient, $k_p$ is permeability, $\phi$ is porosity, $\rho c_p$ is heat capacity, *k* is thermal conductivity, *T* is the temperature of Casson fluid, and $Q_0$ is heat injection/suction term. The corresponding initial conditions are given as

9 $$\begin{aligned} u(\xi ,0)&=0, \quad T(\xi ,0)=T_\infty , \nonumber \\ \xi \ge 0: \quad u_\tau (\xi ,0)&=0, \quad u_\xi (\xi ,0)=0. \end{aligned}$$

The corresponding boundary conditions are given as

10 $$\begin{aligned} u(0,\tau )&=\left\{ \begin{array}{ll} u_c\frac{\tau }{\tau _0} \quad 0 < \tau \le \tau _0 \\ u_c \qquad \qquad \tau >\tau _0 \end{array}\right. , \qquad \qquad \qquad \end{aligned}$$

11 $$\begin{aligned} T(0,\tau )&=\left\{ \begin{array}{ll} T_\infty +(T_w-T_\infty )\frac{\tau }{\tau _0} \quad 0 < \tau \le \tau _0 \\ T_w \qquad \qquad \qquad \qquad \qquad \tau >\tau _0 \end{array}\right. , \end{aligned}$$

12 $$\begin{aligned} \tau >0: \quad u(\xi ,\tau )&\rightarrow 0, \quad T(\xi ,\tau )\rightarrow T_\infty \quad \text {for} \quad \xi \rightarrow \infty . \end{aligned}$$

The choice of an optically thick fluid, which absorbs and emits but does not shatter thermal radiation allows us to use Rosseland approximation for radiative flux. Implementation of Rosseland approximation leads to the following expression for radiative heat flux^49^

13 $$\begin{aligned} Q_r=-\frac{4 \sigma _1}{3 k_1}\frac{\partial T^4}{\partial \xi }, \end{aligned}$$

where $k_1$ and $\sigma _1$ denote the coefficient of Rosseland absorption and Stefan–Boltzmann constant respectively. This non-linear relation of radiative flux can be linearized by assuming that temperature differences are very small. Applying Taylor series to expand $T^4$ about $T_\infty$ and emitting higher-order terms by virtue of the above-mentioned supposition yields the following relation

14 $$\begin{aligned} T^4 \approx 4 T^3_{\infty } T- 3 T^4_{\infty }. \end{aligned}$$

Plugging Eqs. (13) and (14) in Eq. (8) yields

15 $$\begin{aligned} \rho c_p \frac{\partial T}{\partial \tau }=k\left( 1+\frac{16 \sigma _1 T^3_\infty }{3 k_1 k} \right) \frac{\partial ^2 T}{\partial \xi ^2}+Q_0 (T-T_\infty ). \end{aligned}$$

Introducing the following variables

16 $$\begin{aligned} \xi ^*=\frac{\xi u_c}{\nu }, \quad u^*=\frac{u}{u_c}, \quad \tau ^*=\frac{\tau u_c^2}{\nu }, \quad \tau _0=\frac{\nu }{u_c^2}, \quad \theta =\frac{T-T_\infty }{T_w-T_\infty }, \end{aligned}$$

into Eqs. (7) and (15) and dropping * for the purpose of brevity yields

17 $$\begin{aligned} \frac{\partial u}{\partial \tau }&=\left( 1+\frac{1}{\alpha } \right) \frac{\partial ^2 u}{\partial \xi ^2}-\left\{ M+\frac{1}{K} \left( 1+\frac{1}{\alpha } \right) \right\} u+Gr \theta , \end{aligned}$$

18 $$\begin{aligned} \frac{\partial \theta }{\partial \tau }&=\left( \frac{1+Nr}{Pr}\right) \frac{\partial ^2 \theta }{\partial \xi ^{2}}+Q\theta . \qquad \qquad \end{aligned}$$

The pertinent initial and boundary conditions in Eqs. (9)–(12) take the following form

19 $$\begin{aligned} u(\xi ,0)&=0, \quad \theta (\xi ,0)=0, \nonumber \\ \xi \ge 0: \quad u_\tau (\xi ,0)&=0, \quad u_\xi (\xi ,0)=0, \end{aligned}$$

20 $$\begin{aligned} u(0,\tau )&=\theta (0,\tau ) =\left\{ \begin{array}{ll} \tau \qquad 0 < \tau \le 1\\ 1 \qquad\qquad \tau >1 \end{array} \right. , \end{aligned}$$

21 $$\begin{aligned} \tau >0: \quad u(\xi ,\tau )&\rightarrow 0, \quad \theta (\xi ,\tau )\rightarrow 0 \quad \text {when} \quad \xi \rightarrow \infty , \end{aligned}$$

with

22 $$\begin{aligned} Q=\frac{Q_0\nu }{\rho c_p u^2_c}, \quad Gr=\frac{ \nu \beta g (T_w-T_\infty )}{u^3_c}, \quad Pr=\frac{\mu c_p}{k}, \nonumber \\ \frac{1}{K}=\frac{\phi \nu ^2 }{k_p u^2_c}, \quad Nr=\frac{16\sigma _1 T_\infty ^3}{3kk_1}, \quad M=\frac{\sigma B_0^2 \nu }{\rho u_c^2}, \end{aligned}$$

where *Q* is heat injection/suction parameter, *Gr* is Grashof number, *Pr* is Prandtl number, *K* is the parameter of porosity, *Nr* is radiation parameter, and *M* is the magnetic parameter.

## Analytical solutions

The solution of the current problem can be computed conveniently using Laplace transformation due to its efficient applications for nonuniform boundary conditions. The formulation of the integral form of Laplace transform pair for dimensionless temperature and velocity are respectively given as follows

23 $$\begin{aligned} \bar{\theta }(\xi ,q)=\int \limits _{0}^{\infty } e^{-q \tau }\theta (\xi ,\tau )d\tau =\mathcal {L}[\theta ](\tau ), \end{aligned}$$

24 $$\begin{aligned} \bar{u}(\xi ,q)=\int \limits _{0}^{\infty } e^{-q \tau }u(\xi ,\tau )d\tau =\mathcal {L}[u](\tau ). \end{aligned}$$

Using the definition of Laplace transform from Eq. (23) on energy Eq. (18) and relevant boundary conditions (19)$_2$–(21)$_2$, we get

25 $$\begin{aligned}&\frac{d ^2 \bar{\theta }}{d \xi ^2}-c_1 (q-Q) \bar{\theta }=0, \qquad \end{aligned}$$

26 $$\begin{aligned}&\left\{ \begin{array}{ll} \bar{\theta }(0,q)=\frac{1-e^{-q}}{q^2}, \\ \bar{\theta }(\xi ,q)\rightarrow 0 \quad \text {for} \quad \xi \rightarrow \infty , \end{array}\right. \end{aligned}$$

where

$$\begin{aligned} c_1=\frac{Pr}{1+Nr}. \end{aligned}$$

The solution of second order ordinary differential Eq. (25) subject to boundary conditions (26) is calculated as

27 $$\begin{aligned} \bar{\theta }(\xi ,q)=\left( \frac{1-e^{-q}}{q^2}\right) e^{-\sqrt{c_1(q-Q)} \xi }. \end{aligned}$$

In order to find the solution of the velocity field, application of Laplace transform given in Eq. (24) on Eq. (17) and relevant boundary conditions (19)$_1$–(21)$_1$ yields

28 $$\begin{aligned}&\frac{d^2 \bar{u}}{d \xi ^2}-\left( \frac{q+c_3}{c_2} \right) \bar{u}=-\frac{Gr}{c_2}\bar{\theta }, \end{aligned}$$

29 $$\begin{aligned}&\left\{ \begin{array}{ll} \bar{u}(0,q)=\frac{1-e^{-q}}{q^2}, \\ \bar{u}(\xi ,q)\rightarrow 0 \quad \text {for} \quad \xi \rightarrow \infty . \end{array}\right. \end{aligned}$$

Plugging the value of $\bar{\theta }$ from Eq. (27) in Eq. (28) turns out as

30 $$\begin{aligned} \frac{d^2 \bar{u}}{d \xi ^2}-\left( \frac{q+c_3}{c_2} \right) \bar{u}=-\frac{Gr}{c_2} \left( \frac{1-e^{-q}}{q^2}\right) e^{-\sqrt{c_1(q-Q)} \xi }. \end{aligned}$$

The solution of Eq. (30) subject to boundary conditions (29) is acquired as

31 $$\begin{aligned} \bar{u}(\xi ,q)=\left( \frac{1-e^{-q}}{q^2}\right) \bar{H}(\xi ,q), \end{aligned}$$

where

32 $$\begin{aligned} \bar{H}(\xi ,q)=e^{-\sqrt{\frac{q+c_3}{c_2}}\xi } +\frac{Gr e^{-\sqrt{\frac{q+c_3}{c_2}}\xi }}{(c_1 c_2-1)(q-c_4)}-\frac{Gr e^{-\sqrt{c_1(q-Q)} \xi }}{(c_1 c_2-1)(q-c_4)}, \end{aligned}$$

with

33 $$\begin{aligned} c_2=1+\frac{1}{\alpha }, \quad c_3= M+\frac{1}{K} c_2, \quad c_4=\frac{c_3+ c_1 c_2 Q}{c_1 c_2-1}. \end{aligned}$$

**Figure 2:**
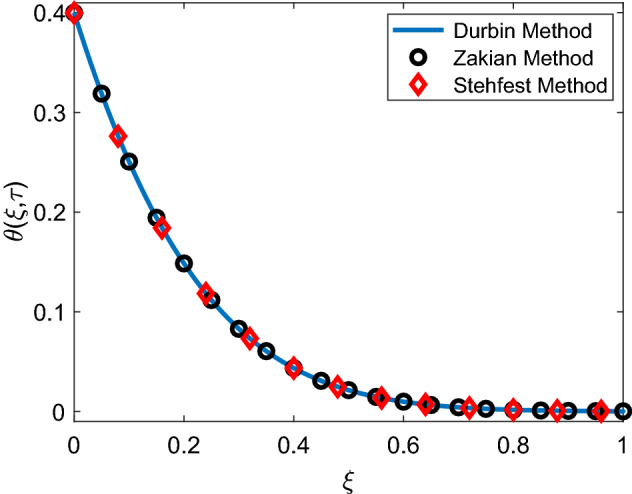
Validation of temperature solution.

**Figure 3:**
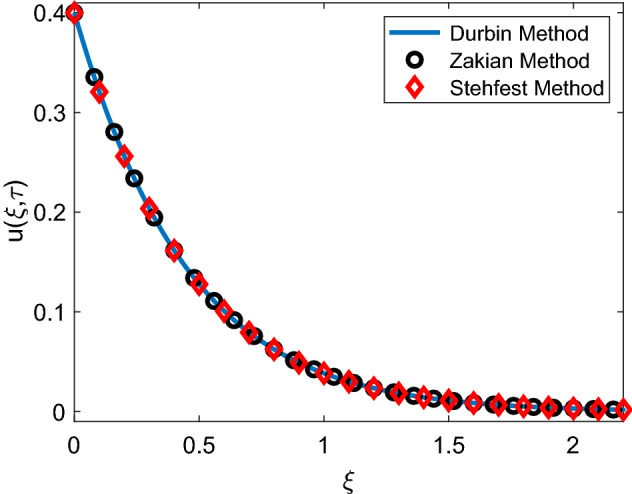
Validation of velocity solution.

It is obvious to observe that Laplace domain solutions of temperature field (27) and velocity field (31) are comprised of multivalued relations of Laplace parameter *q*, therefore to compute the inverse Laplace transformation by handling these complex combinations efficiently, we implemented the Durbin method given in^50^. Additionally, these velocity and energy solutions are validated in Figs. 2 and 3 by drawing a comparison with Zakian and Stehfest numerical Laplace inversion methods^51,52^.

## Limiting models

Some special cases of our current problem are discussed here to examine the effects of the absence of some particular parameters on solutions.

### Solution in the absence of porosity and MHD

Taking $M=\frac{1}{K}=0$ in Eq. (31), it reduces to

34 $$\begin{aligned} u(\xi ,\tau )=\mathcal {L}^{-1} \left[ \left\{ \frac{1-e^{-q}}{q^2}\right\} \left\{ e^{-\sqrt{\frac{q}{c_2}}\xi } +\frac{Gr e^{-\sqrt{\frac{q}{c_2}}\xi }}{(c_1 c_2-1)\left( q-\frac{c_1 c_2 Q}{c_1 c_2-1} \right) } \right. \right. \nonumber \\ \left. \left. -\frac{Gr e^{-\sqrt{c_1(q-Q)} \xi }}{(c_1 c_2-1)\left( q-\frac{c_1 c_2 Q}{c_1 c_2-1}\right) } \right\} \right] . \qquad \end{aligned}$$

### Solution in the absence of Casson parameter

Taking $\frac{1}{\alpha }=0$ in Eq (31), it turn out as

35 $$\begin{aligned} u(\xi ,\tau )=\mathcal {L}^{-1} \left[ \left\{ \frac{1-e^{-q}}{q^2}\right\} \left\{ e^{-\sqrt{q+m}\xi } +\frac{Gr e^{-\sqrt{q+m}\xi }}{(c_1 -1)\left( q-\frac{m+ c_1 Q}{c_1 -1}\right) } \right. \right. \nonumber \\ \left. \left. -\frac{Gr e^{-\sqrt{c_1(q-Q)} \xi }}{(c_1-1)\left( q-\frac{m+ c_1Q}{c_1-1}\right) } \right\} \right] , \qquad \end{aligned}$$

where $$m=M+\frac{1}{K}$$.

### Solution in the absence of thermal radiation and heat injection/suction parameters

Taking $Nr=Q=0$ in our problem also affects the temperature solution therefore, Eqs. (27) and (31) come out as

36 $$\begin{aligned} \theta (\xi , \tau )&=F(\xi , \tau )-F(\xi , \tau -1) G(\tau -1), \qquad \qquad \end{aligned}$$

37 $$\begin{aligned} u(\xi ,\tau )&=\mathcal {L}^{-1} \left[ \left\{ \frac{1-e^{-q}}{q^2}\right\} \left\{ e^{-\sqrt{\frac{q+c_3}{c_2}}\xi } +\frac{Gr e^{-\sqrt{\frac{q+c_3}{c_2}}\xi }}{(Pr c_2-1)\left( q-\frac{c_3}{Pr c_2-1}\right) } \right. \right. \nonumber \\&\quad \left. \left. -\frac{Gr e^{-\sqrt{Pr q} \xi }}{(Pr c_2-1)\left( q-\frac{c_3}{Pr c_2-1}\right) } \right\} \right] , \qquad \end{aligned}$$

where

$$\begin{aligned} F(\xi , \tau )=\left( \tau +\frac{Pr \xi ^2}{2} \ \right) {\text {erfc}}\left( \frac{\xi \sqrt{Pr}}{2 \sqrt{\tau }} \right) -\frac{\xi \sqrt{Pr \tau }}{\sqrt{\pi }} e^{- \frac{\xi ^2 Pr}{4 \tau }}, \end{aligned}$$

and $G(\tau -1)$ is presenting a Heaviside function.

### Solution for constant velocity and constant temperature conditions

The temperature and velocity solutions of Casson fluid for isothermal wall temperature and constant wall velocity are evaluated as

38 $$\begin{aligned} \theta (\eta , \tau )&=e^{-\xi \dot{\imath } \sqrt{c_1 Q}} {\text {erfc}} \left( \frac{\xi \sqrt{Q}}{2 \sqrt{\tau }}-\dot{\imath } \sqrt{Q \tau } \right) +e^{\xi \dot{\imath } \sqrt{c_1 Q}} {\text {erfc}} \left( \frac{\xi \sqrt{Q}}{2 \sqrt{\tau }}+\dot{\imath } \sqrt{Q \tau } \right) , \end{aligned}$$

39 $$\begin{aligned} u(\xi ,\tau )&=\mathcal {L}^{-1} \Bigg [\left\{ \frac{1}{q}\right\} \Bigg \{ e^{-\sqrt{\frac{q+c_3}{c_2}}\xi } +\frac{Gr e^{-\sqrt{\frac{q+c_3}{c_2}}\xi }}{(c_1 c_2-1)\left( q-c_4\right) } \qquad \Bigg . \Bigg . \nonumber \\&\quad \Bigg . \Bigg . -\frac{Gr e^{-\sqrt{c_1(q-Q)} \xi }}{(c_1 c_2-1)(q-c_4)} \Bigg \}\Bigg ]. \qquad \qquad \end{aligned}$$

### Solution of Newtonian fluid for constant wall velocity and ramped wall temperature conditions

The ramped wall temperature and constant wall velocity profiles of a Newtonian fluid ($\frac{1}{\alpha }=0$) can be calculated as

40 $$\begin{aligned} \theta (\xi ,\tau )&=\mathcal {L}^{-1}\left[ \left( \frac{1-e^{-q}}{q^2}\right) e^{-\sqrt{c_1(q-Q)} \xi }\right] , \end{aligned}$$

41 $$\begin{aligned} u(\xi , \tau )&=\mathcal {L}^{-1}\bigg [\frac{1}{q}e^{-{\sqrt{q+m}\xi }} +\left( \frac{Gr}{q(c_1-1)-(c_1 Q+m)}\frac{1-e^{-q}}{q^2}\right) \times \bigg . \nonumber \\&\quad \bigg . \left( e^{-\sqrt{q+m}\xi }-e^{-\sqrt{c_1(q-Q)} \xi } \right) \bigg ], \end{aligned}$$

where $$m=M+\frac{1}{K}$$.

## Skin friction and Nusselt number

The relations for skin friction $C_f$ (wall shear stress) and Nusselt number Nu are presented as

42 $$\begin{aligned} C_f&=\left( 1+\frac{1}{\alpha }\right) \frac{d u(\xi ,\tau )}{d \xi } \bigg |_{\xi =0}, \end{aligned}$$

43 $$\begin{aligned} Nu&=-\frac{d \theta (\xi ,\tau )}{d \xi }\bigg |_{\xi =0}, \end{aligned}$$

where

44 $$\begin{aligned} \frac{d u(\xi ,\tau )}{d \xi }\bigg |_{\xi =0}&=\mathcal {L}^{-1}\left[ \left( \frac{1-e^{-q}}{q^2}\right) \bar{H}_1(\xi ,q) \right] , \end{aligned}$$

45 $$\begin{aligned} \frac{d \theta (\xi ,\tau )}{d \xi }\bigg |_{\xi =0}&=\mathcal {L}^{-1}\left[ \left( \frac{1-e^{-q}}{q^2}\right) \sqrt{c_1(q-Q)} \right] , \end{aligned}$$

with

46 $$\begin{aligned} \bar{H}_1(\xi ,q)=\frac{Gr \sqrt{c_1(q-Q)}}{(c_1 c_2-1)(q-c_4)}-\frac{Gr \sqrt{\frac{q+c_3}{c_2}}}{(c_1 c_2-1)(q-c_4)}-\sqrt{\frac{q+c_3}{c_2}}. \end{aligned}$$

## Parametric study

This section covers noteworthy features of several connected parameters like Grashof number (*Gr*), porosity parameter (*K*), magnetic parameter (*M*), Casson parameter ($\alpha$), Prandtl number (*Pr*), radiation parameter (*Nr*), heat injection/suction parameter (*Q*), and time ($\tau$) on dimensionless flow and energy profiles. The numerical computations are categorized into two divisions: (1) Casson fluid with ramped wall velocity and ramped wall temperature (represented by solid lines) and (2) Casson fluid with constant wall velocity and constant wall temperature (represented by dashed lines). Furthermore, the impacts of the aforementioned parameters on Nusselt number and skin friction are observed with the aid of computed results provided in Tables 1 and 2.

**Table 1:** Variation of Nusselt number for different values of parameters.

${\tau }$	${Pr}$	${Nr}$	${Q}$	${Nu}$
**0.4**	21.0	2.0	0.5	1.7595
**0.5**	–	–	–	1.9304
**0.6**	–	–	–	2.0738
0.7	**0.7**	–	–	0.4008
–	**7.0**	–	–	1.2675
–	**21.0**	–	–	2.1954
–	21.0	**1**	–	2.6888
–	–	**2**	–	2.1954
–	–	**3**	–	1.9013
–	–	2.0	**− 1.0**	3.0433
–	–	–	**− 0.5**	2.7793
–	–	–	**0.0**	2.4976
–	–	–	**0.5**	2.1954
–	–	–	**1.0**	1.8694

Bold values are used to specify the variation of a particular parameter.

**Table 2:** Variation of skin friction for different values of parameters.

${\tau }$	${M}$	${K}$	${\alpha }$	${Gr}$	${C_f}$
**0.4**	2.0	0.5	1.0	1.0	− 1.7444
**0.5**	–	–	–	–	− 2.1051
**0.6**	–	–	–	–	− 2.4621
0.7	**1.0**	–	–	–	− 2.2825
–	**2.0**	–	–	–	− 2.711
–	**3.0**	–	–	–	− 2.6484
–	2.0	**0.3**	–	–	− 3.2248
–	–	**0.6**	–	–	− 2.7052
–	–	**0.9**	–	–	− 2.5114
–	–	0.5	**0.2**	–	− 6.9092
–	–	–	**0.5**	–	− 3.8643
–	–	–	**0.9**	–	− 2.9343
–	–	–	1.0	**1.0**	− 2.9329
–	–	–	–	**3.0**	− 2.6989
–	–	–	–	**5.0**	− 2.4649

Bold values are used to specify the variation of a particular parameter.

**Figure 4:**
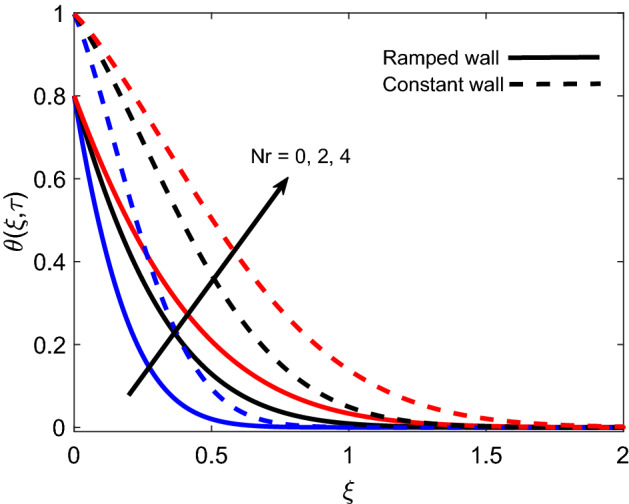
Energy distribution for various values of *N*r.

**Figure 5:**
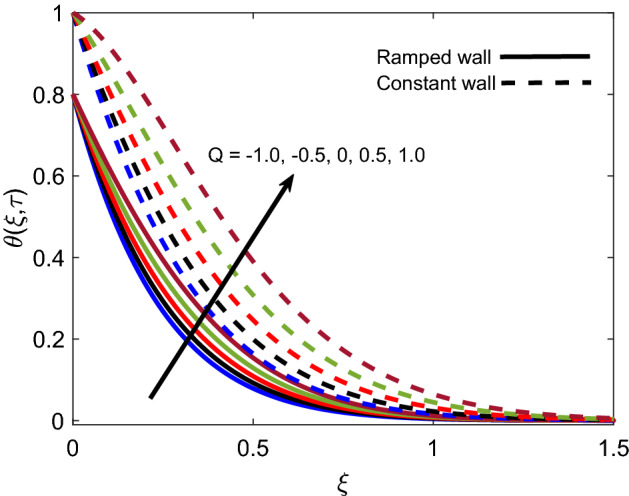
Energy distribution for various values of *Q*.

Figure 4 highlights the impact of *Nr* on the energy profile of Casson fluid. It is noticed that temperature receives elevation with an increase in values of *Nr*. Physically, along normal direction to plate, change of heat flux $\frac{\partial Q_r}{\partial \xi }$ rises and $k_1$ reduces, which implies that more amount of radiative heat is transferred to the fluid and consequently temperature profile rises. The temperature profile is found higher in the case of simultaneous constant boundary conditions. Figure 5 presents the relationship between temperature and the amount of heat either injected ($Q>0$) or sucked ($Q<0$). The figure shows that when positive *Q* increases temperature profile also increases since this increment corresponds to an increase in the amount of injected heat. On the other hand, temperature faces a decay when the magnitude of *Q* increases in a negative direction because this increment implies that the amount of heat released by the fluid is increasing. Subsequently, this explanation justifies the physical logic and highlights the significance of heat generation/suction in heating and cooling processes.

**Figure 6:**
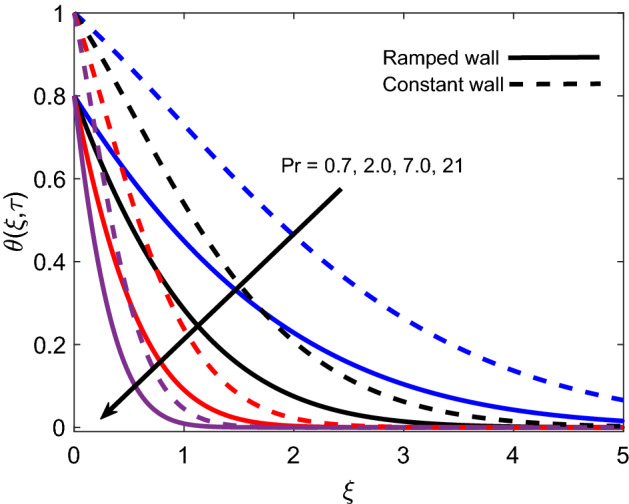
Energy distribution for various values of *Pr*.

**Figure 7:**
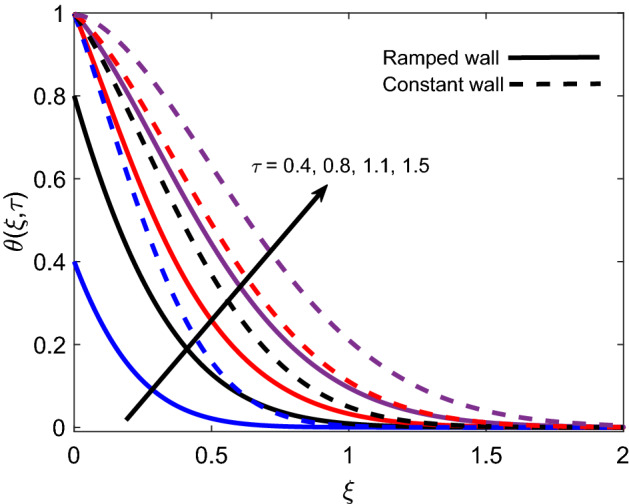
Energy distribution for various values of $\tau$.

The distribution of temperature for various *Pr* values is presented in Fig. 6 for both ramped wall condition and isothermal wall condition. For both cases, it is spotted that the temperature profile declines as *Pr* grows. It is physically certified by the fact that fluid with a higher *Pr* value has comparatively less thermal conductivity, which minimizes the conduction of heat. As a result, the thickness of the thermal boundary layer shrinks. Ultimately, the temperature of fluid reduces. Figure 7 illustrates that the temperature of Casson fluid is an increasing function of $\tau$. Additionally, the temperature is greater in the case of ramped temperature condition in contrast to isothermal wall condition. The temperature near the plate has higher values and it calms down asymptotically to zero-value as fluid flows far away from the wall.

**Figure 8:**
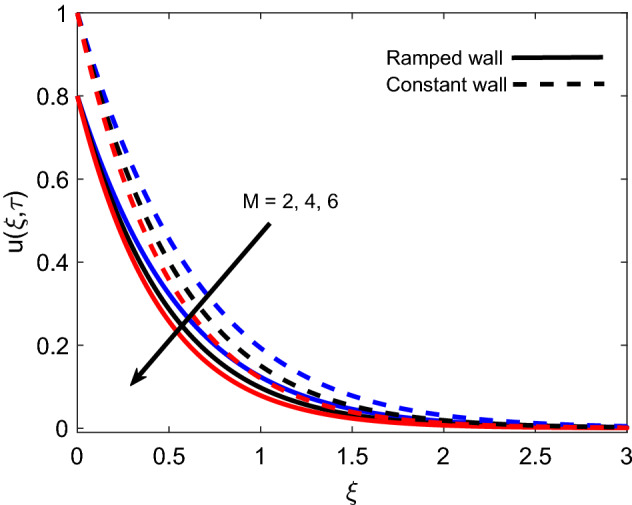
Velocity distribution for various values of *M*.

**Figure 9:**
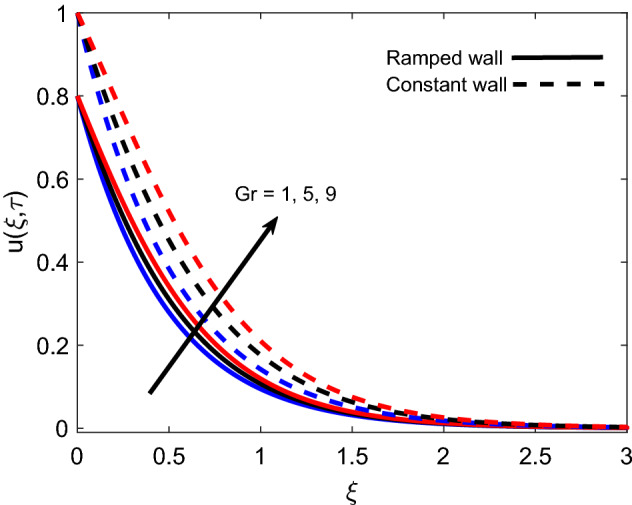
Velocity distribution for various values of *Gr*.

**Figure 10:**
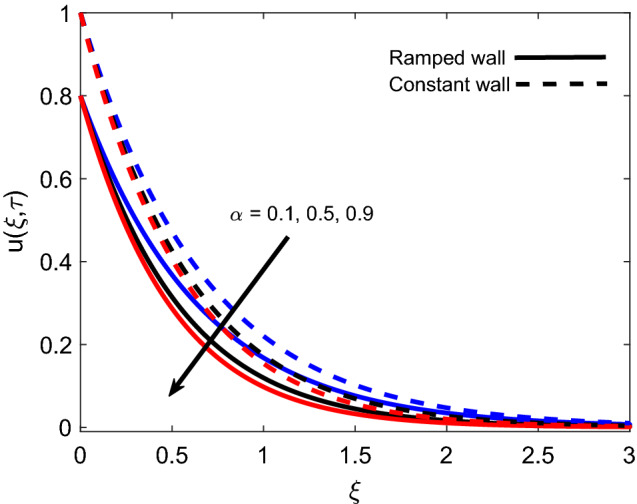
Velocity distribution for various values of $\alpha$.

The influence of *M* on the velocity profile for ramped and isothermal wall conditions is revealed in Fig. 8. It is sighted that an increase in strength of the magnetic field reduces both magnitude of velocity and boundary layer thickness. This is due to the fact that the imposition of the magnetic field results in the establishment of a strong Lorentz force, which acts as a dragging force and offers resistance to fluid flow. Eventually, fluid gets decelerated with an increase in *M* because dragging force dominates the flow supporting forces. Furthermore, the velocity profile has a relatively greater elevation in the case of isothermal condition in contrast to the ramped condition. Figure 9 covers the contribution of *Gr* in fluid flow for ramped plate and isothermal plate. Both flow profiles depict that enhancement in *Gr* is a favorable factor as it accelerates the flow in both cases. The physical logic justifying this behavior is the strengthening of thermal buoyancy force. Since *Gr* deals with the fraction of buoyancy force and viscous force, an increase in *Gr* implies that buoyancy force suppresses the viscous effects, which leads to reduce the offered resistance. Hence, fluid gets accelerated near the plate and far away from the plate, it calms down as buoyancy force together with associated forces gets weaker. Figure 10 exhibits correspondence of the velocity profile and Casson parameter $\alpha$. It is observed that they share an inverse relation, as an increase in $\alpha$ results in flow retardation. The physical phenomenon countering this retardation is the plasticity of fluid. When parameter $\alpha$ reduces, momentum boundary layer thickness increases due to an increase in the plasticity of fluid. It is also witnessed that $\alpha$ has a similar effect on both ramped wall and isothermal wall solutions. Furthermore, it is worthy to mention that when $\alpha$ is very large ($\frac{1}{\alpha } \rightarrow 0$), the non-Newtonian behavior of fluid fully vanishes, and fluid reduces to a purely Newtonian fluid.

**Figure 11:**
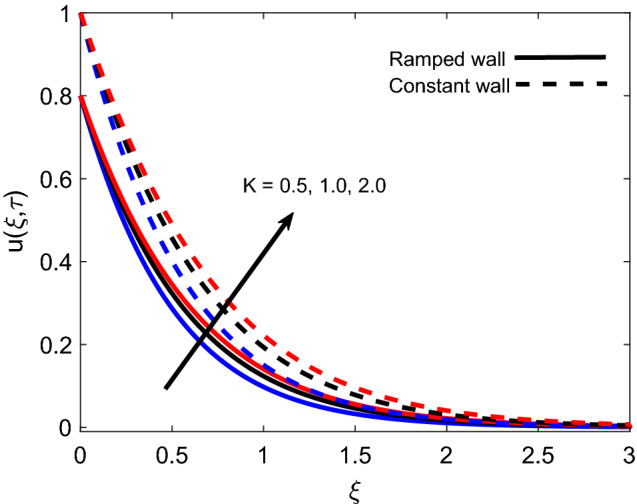
Velocity distribution for various values of *K*.

**Figure 12:**
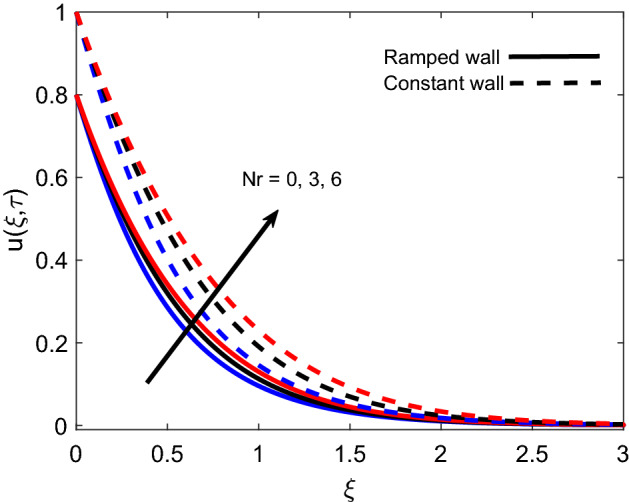
Velocity distribution for various values of *Nr*.

**Figure 13:**
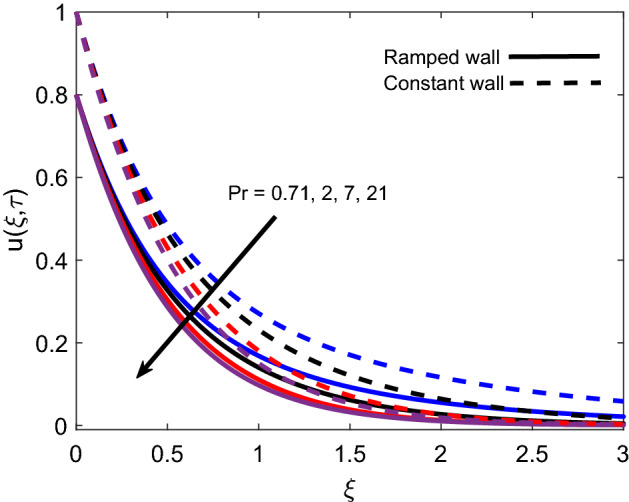
Velocity distribution for various values of *Pr*.

The variation in isothermal wall momentum distribution and ramped wall momentum distribution for various values of *K* is reported in Fig. 11. The figure exhibits that larger values of *K* escalate the fluid velocity and boundary layer thickness, which is justified by the physical logic that an increase in the porosity of media reduces the strength of the resistive force and consequently, momentum development enhances in flow regime. Moreover, velocity is found lower for ramped conditions against isothermal conditions. Figure 12 discloses the effect of variation in *Nr* on flow profile and it is spotted that velocity enhances for larger values of *Nr*. This is authenticated by the logic that thermal radiation heats up the fluid and this higher rate of energy transfer looses the bonds between fluid particles. Successively, the offered resistance gets weaker and flow is accelerated. Velocity profiles for ramped wall and isothermal wall follow similar trends for thermal radiation. Figure 13 interprets the influence of *Pr* on momentum profile. Ramped wall and isothermal wall flows are compared, and it is observed that later flow has faster velocity. For increasing variation of *Pr*, velocity profile behaves inversely, which is physically supported by an intensification of momentum diffusivity. As a result, dragging force dominates near the plate and fluid faces more resistance. Far away from the plate, dragging force gradually decreases and fluid comes to rest.

Table 1 presents that the Nusselt number is an increasing function of *Pr* and $\tau$ while it behaves oppositely for *Nr* and *Q*. An important observation is made here that initially heat transfer rate enhances for $\tau <1$ and later it starts decreasing for $\tau >1$. Table 2 provides that the wall shear stress reduces with elevation in $\tau$ and *M*, and it augments for increasing values of *K*, *Gr*, and $\alpha$. For both tables, in each row, one column encloses bold values for a particular parameter to focus on the role of that parameter in heat transfer and skin friction.

## Conclusion

The main aim of this study is to examine the physical features of combined ramped velocity condition and ramped temperature condition on unsteady Casson fluid flow over an infinitely long vertical plate. The plate is nested in a porous media and a uniform magnetic force is applied. Besides, heat injection/suction and thermal radiation are also included in the model. Some appropriate transformations are employed for the sake of non-dimensionalization of principal equations, and later Laplace transformation is operated to compute the solutions. Combined ramped conditions (ramped wall velocity and ramped wall temperature) are practically significant though their analytical handling leads to complicated relations. Hence, results in basic coordinates are computed through numerical Laplace inversion called the Durbin method. These results are validated with two more numerical inversion methods named Zakian and Gaver-Stehfest methods. The control of connected parameters on dimensionless temperature and velocity solutions is graphically elaborated. Meanwhile, the computed results for Nusselt number and skin friction are reported through tables. A comparison between solutions with ramped conditions and solutions with isothermal conditions is also drawn graphically. The prime observations of this investigation are remarked as

- Fluid velocity is a decreasing function of the Casson parameter $\alpha$.
- An increase in Prandtl number and heat suction reduce the temperature of the fluid.
- Porosity and magnetic parameter have a relatively inverse influence on momentum boundary layer thickness.
- Velocity on the wall can be handled with larger values of $\alpha$ (connected to skin friction).
- Heat transfer rate is minimized when radiation parameter escalates, while a decline in thermal diffusivity implies a decrease in heat transfer rate. Accordingly, the heat transfer process can be controlled or supported by larger and smaller values of Prandtl number (associated to Nusselt number)^53,54^.

## Data Availability

All the relevant material is available.
